# Equal representation or federal equity? Constitutional challenges of India’s post-2026 delimitation under Article 82

**DOI:** 10.3389/fsoc.2026.1884362

**Published:** 2026-09-24

**Authors:** Kavali Munisekhar, Bangalore Morarji

**Affiliations:** School of Social Sciences and Languages, Vellore Institute of Technology, Vellore, India

**Keywords:** comparative federalism, delimitation commission, electoral redistricting, Indian federalism, malapportionment, women’s political reservation

## Abstract

When India’s five-decade freeze on legislative seat allocation expires with the 2026 census, the ensuing delimitation will force a choice the Constitution has so far deferred: between the equal-suffrage command of “one person, one vote” and the federal-equity claims of states that curbed their populations and now face reduced representation. This paper asks how that choice should be made, and whether the government’s proposal to expand the Lok Sabha by roughly half resolves it. Combining doctrinal legal analysis with an illustrative, reproducible seat-allocation model and a comparison with four mature federations, it advances two arguments. First, India is distinctive among large federations in lacking a structural counterweight to population unlike the second chambers of Australia, Canada, Germany, and the United States, its Rajya Sabha is itself largely population-based, so the entire North–South tension loads onto Lok Sabha delimitation. Second, seat expansion is necessary but not sufficient: it prevents absolute seat loss but not relative marginalisation, and a durable settlement requires either a weighted-equity allocation or a rebalanced second chamber, each achievable only by constitutional amendment. The paper specifies such a weighted formula, tests it against the equal-suffrage guarantee of Article 81, and assesses the political and institutional feasibility of the alternatives. It contributes a comparative diagnosis of India’s federal-equity deficit and a substantiated, feasibility-tested reform pathway for one of the most consequential constitutional questions of the post-2026 period.

## 1 Introduction

The structural foundation of any representative democracy is constituency delimitation, the periodic redrawing of electoral boundaries so that representation keeps pace with population change (Patel and Sekher, 2026). This is especially significant in India, the world’s most populous democracy (Alam and Sivaramakrishnan, 2015). A population of more than 1.4 billion spread across 28 states and 8 Union Territories, with remarkable linguistic, religious, and demographic diversity, makes the way electoral constituencies are drawn a question not only of the composition of Parliament but of the practical operation of federalism, the equality of individual voting rights, and the representational efficacy of historically marginalised communities (Varshney, 2000). To manage this task, the Delimitation Commission of India was created as a quasi-judicial body under the Delimitation Commission Acts of 1952, 1962, 1972, and 2002. Chaired by a retired Supreme Court judge, with the Chief Election Commissioner as an ex-officio member (Rudolph and Rudolph, 1987), its decisions are constitutionally final: they cannot be challenged in any court and Parliament cannot amend them. This institutional design is a deliberate attempt to insulate boundary-drawing from party influence—the phenomenon known in comparative electoral study as gerrymandering (Singh, 2000; Gudgin and Taylor, 1979; Lublin, 2020).

The history of delimitation in India is nonetheless a narrative of both extended interruption and periodic change (Sivaramakrishnan, 2000). The most significant suspension came in 1976, when the 42nd Constitutional Amendment, passed during the Emergency, froze the total number of assembly and parliamentary seats allotted to each state at the levels determined by the 1971 census (Kumar, 2018). The stated objective was to protect states that had conducted population-control programmes from losing representation (McMillan, 2001; Hussain, 2008). The 84th Amendment of 2001 prolonged the freeze until the first census after 2026, solidifying a demographic foundation that has now been in place for more than 50 years (Vaishnav and Hintson, 2019; Verma, 2002).

The year 2026 marks the official termination of this freeze. Census 2026 began on 1 April 2026, with complete data anticipated by 2027 (Patel and Sekher, 2026). Concurrently, the government has announced a Constituency Delimitation Bill, to be presented in a special parliamentary session, that aims to increase the number of Lok Sabha seats by roughly 50%, from 543 to about 816, and to activate the 33% women’s reservation mandated by the Nari Shakti Vandan Adhiniyam, 2023 (De Santo and Le Maux, 2023). This combination of constitutional duty, demographic pressure, and political calculation has provoked one of the most important public-law debates in India’s post-independence history.

### 1.1 The constitutional dilemma of post-2026 delimitation

The 2026 delimitation presents not a technical apportionment problem but a constitutional dilemma between equal individual suffrage and federal equity, one that seat expansion alleviates but cannot resolve, and whose durable settlement requires India to acquire, by constitutional amendment, the kind of federal-equity counterweight that comparable federations locate in a second chamber or in statutory seat-floors.

### 1.2 Research gap

Existing scholarship approaches delimitation from separate directions: the constitutional history of the freeze (Sivaramakrishnan, 2000; McMillan, 2001; Kumar, 2018), empirical projection of the North–South seat shift (Vaishnav and Hintson, 2019; Patel and Sekher, 2026), and the measurement of malapportionment (Daxecker, 2020). What is missing is a treatment that integrates the legal, federal, demographic, geographic, and sociological dimensions in one framework, subjects the proposed remedies to a reproducible allocation model rather than assertion, and situates them within comparative federal practice and political feasibility. This paper addresses that gap, contributing a comparative diagnosis of India’s federal-equity deficit and a substantiated, feasibility-tested reform pathway.

The article is organised in nine sections: the conceptual framework (Section 2); methodology (Section 3); the constitutional and legal architecture (Section 4); the historical evolution of the delimitation commissions (Section 5); recent delimitation exercises (Section 6); the 2026 political economy, including comparative federal perspectives (Section 7); key challenges and reform proposals, including implementation and alternatives (Section 8); and the conclusion (Section 9).

## 2 Conceptual framework

This paper’s conceptual framework combines five theoretical traditions that together explain the multifaceted nature of delimitation as a governance problem. Figure 1 illustrates how these perspectives focus on the Delimitation Commission as an institution, move through the historical stages of constitutional modification, and produce the current conflicts that reform must resolve.

**Figure 1:**
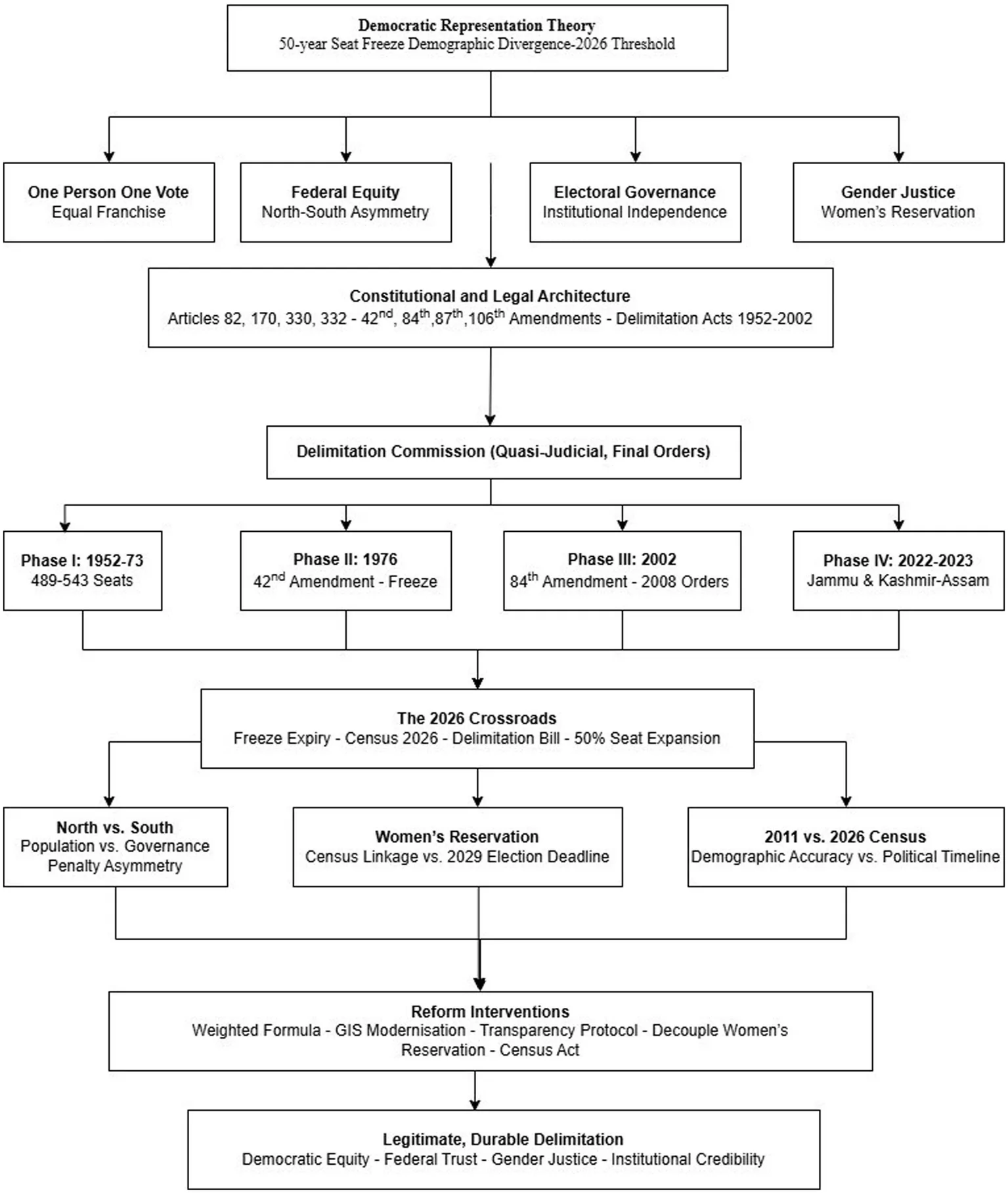
Conceptual framework.

These five lenses are not parallel silos but a connected sequence, each generating the question the next must answer. The one-person-one-vote lens sets the normative baseline, equal suffrage against which any deviation must be justified. The federalism lens supplies the justification others offer for deviating: that sub-national equity, and the “population-control penalty,” warrant weighting representation away from raw numbers. The electoral-governance lens then asks whether the institutional process that draws the boundaries is itself legitimate enough to be trusted with such deviations, a question the case studies test directly. The gender-justice lens tracks a distributional consequence that rides on the same exercise, since the 106th Amendment’s reservation is triggered by this delimitation. The social-cleavage lens, finally, follows the consequences down to the constituency level, where boundaries reshape caste and community representation. Empirically, each lens fixes a question applied to the same materials, and the reform proposals are assessed against all five simultaneously, which prevents a fix for one dimension from silently worsening another.

### 2.1 Democratic representation theory: one person, one vote

The fundamental rationale for periodic delimitation stems from the theory of “one person, one vote, one value” (OPOV), which holds that every vote in a democratic society should carry equal weight in determining electoral outcomes (Norris, 2004; Daxecker, 2020). The OPOV principle, articulated for the United States in *Reynolds v. Sims* (1964) and recognised in the comparative electoral-law literature as a component of democratic legitimacy, holds that population differences between constituencies reduce the effective franchise of voters in more populous regions, producing structural inequality in democratic participation (Ansolabehere et al., 2002). Although the Supreme Court of India has permitted substantial departures from OPOV in the name of administrative convenience and federal sensitivity, it has tacitly accepted the principle as a constitutional promise through Articles 81, 82, and 170. The scale of the divergence is considerable: by recent estimates there is roughly one Member of Parliament for every 3.24 million residents in Rajasthan, compared with 1.79 million per Member in Kerala. A systematic disparity of this kind, a passive malapportionment produced by inaction rather than design sits in tension with the guarantee of equality under Article 14 (Rossiter et al., 2009; Horiuchi and Saito, 2003).

### 2.2 Federalism theory: the sub-national representation problem

India’s federal framework presents what federalism scholars term the “population-control penalty”: the perverse outcome in which states that effectively curbed their fertility notably the southern states of Tamil Nadu, Kerala, Karnataka, Andhra Pradesh, and Telangana face diminished Lok Sabha representation under a strictly population-proportional delimitation, while high-fertility northern states gain political power (Sivaramakrishnan, 2000; Srinivasulu, 2024). Applying the Webster method to projected population, Patel and Sekher (2026) estimate a substantial northern gain and southern loss under post-2026 delimitation; a comparable analysis on 2011 Census data by Vaishnav and Hintson (2019) yields a smaller shift, the difference being one of base year rather than direction. This imbalance reflects a deeper conflict in federal theory between the pluralist principle of sub-national equality and the majoritarian principle of proportional representation (Alam and Sivaramakrishnan, 2015). India’s failure to resolve that conflict through allocation formulas weighing both demographic and developmental indices has produced what Vaishnav and Hintson (2019) describe as a growing “crisis of representation” (Tillin, 2013; Suri, 2004).

### 2.3 Electoral governance theory: institutional Independence and process integrity

The study of electoral governance, drawing on Norris (2004) and the broader research on electoral-management bodies, underscores that public perception of the integrity of the boundary-setting process is as constitutionally important as its formal outputs (Bhavnani, 2017; Daxecker, 2020). An institution may issue legally binding directives that nonetheless lack democratic legitimacy if its procedure is thought to be controlled, opaque, or open to administrative manipulation (Nishikawa and Herron, 2004). India’s Delimitation Commission, though it scores highly on formal independence, has been criticised on procedural grounds. The Assam delimitation (2023) showed how executive decisions on administrative boundaries particularly the December 2022 merger of four newly created districts just before delimitation began can influence the Commission’s outputs even when they lie technically outside its purview (Election Commission of India, 2023). Examining the 2002 delimitation in Andhra Pradesh and Rajasthan, Iyer and Reddy (2013) found that the exercise was largely politically neutral overall, with detectable influence only where incumbents sat as advisory members, a finding that locates the manipulation risk precisely rather than assuming it.

### 2.4 Gender justice theory: women’s political representation

The fourth lens examines gender justice in political representation. Following the 2024 elections, women held 13.6% of Lok Sabha seats, placing India roughly 145th worldwide (Inter-Parliamentary Union, 2024; Tripp and Kang, 2008), well below the global average of about 26.5% and the levels attained by Rwanda, the Nordic countries, and several Latin American democracies (Spary, 2020; Bhalotra et al., 2018; Pande, 2003). The Nari Shakti Vandan Adhiniyam, 2023, mandating a 33% reservation for women in the Lok Sabha and state assemblies, is India’s most substantial legislative action on this disparity since the 73rd Amendment (1992) reserved seats for women in Panchayati Raj institutions (Buragohain, 2024; Chattopadhyay and Duflo, 2004). Its implementation, however, was made contingent on the first census after commencement and the subsequent delimitation. This linkage has heightened the political urgency of the 2026 exercise and placed gender justice at risk of being used as an instrument for a broader constitutional restructuring that has not been adequately debated (Klugman and Morton, 2023). Dovi (2007) argues that the legitimacy of gender quotas depends not solely on the legal structure but on the transparency and inclusiveness of the implementation mechanisms (Meier, 2008; Teele et al., 2018).

### 2.5 Social cleavage and identity representation

Delimitation is not only a legal, federal, demographic, and geographic exercise; it is a sociological one. Because a constituency is won by assembling a plurality within a bounded population, where the boundary is drawn determines which social configurations can form electoral majorities. Chandra’s (2004) account of “ethnic head counts” in India’s patronage democracy makes the mechanism explicit: voters and party entrepreneurs condition their strategies on the arithmetic of co-ethnics within the unit, so a redrawn boundary that alters that arithmetic alters the competitive logic of the seat itself. Redistricting therefore does more than reallocate seats between states; within states it redistributes the capacity of caste, linguistic, and religious groups to translate numbers into representation.

Two features of the Indian system sharpen this. First, reserved constituencies for Scheduled Castes and Scheduled Tribes are not fixed in place: each delimitation relocates them to the units where the reserved-population share is highest, moving the seats on which Dalit and Adivasi candidates can or must contest (Jensenius, 2016, 2017). Second, the evidence that reservation delivers substantive rather than merely descriptive representation is mixed, reservation has reduced elite-level discrimination without consistently redirecting policy toward reserved-group constituents (Jensenius, 2016), while Bhavnani (2017) shows that quota effects can persist after a seat is de-reserved. A framework that treats seats as interchangeable population containers cannot see any of this. The fifth lens asks a different question of every boundary: not only how many voters it contains, but which communities of interest it composes or divides, and whose representation it enables or forecloses.

## 3 Methodology

This study uses a mixed doctrinal–analytical design with an illustrative quantitative component. The qualitative core triangulates four techniques: doctrinal legal analysis of the constitutional text and amendments (Articles 81, 82, 170, 327, 330, 332; the 42nd, 84th, 87th, and 106th Amendments); historical-institutional analysis of the four Delimitation Commissions, tracing the path dependencies that constrain present options; structured comparative case analysis of the Jammu & Kashmir (2022) and Assam (2023) delimitations across four fixed dimensions (legal foundation, census reference year, consultative procedure, and political consequence); and comparative-federal analysis of Australia, Canada, Germany, the United States, and the European Union. The Delimitation Commission operates at the intersection of constitutional law, federal politics, and electoral governance, a field in which the most important insights come from the interpretation of texts, procedures, and power dynamics rather than the measurement of variables (Yin, 2018; Weiner, 1989).

To move the weighted-equity proposal beyond assertion, the study adds a reproducible apportionment model applying the Webster/Sainte-Laguë method to state population and development indicators, with a sensitivity analysis across weightings, reported in full in the Supplementary material. Evidence is drawn from three source types selected through purposive sampling: primary legal instruments and Delimitation Commission orders (the Constitution, the four Delimitation Acts, and the J&K and Assam final orders); official statistics (Census of India 2011; Sample Registration System 2021; National Commission on Population projections; the Economic Advisory Council to the Prime Minister; and Finance Commission reports); and peer-reviewed scholarship in electoral law and federalism.

The analysis is interpretive and scenario-based rather than predictive. Two limitations follow and should be stated plainly. First, at the time of writing (2026) the Constituency Delimitation Bill had been announced but not enacted, so the analysis of its operation is necessarily provisional. Second, because the 2026 census data are not yet available and no primary elite fieldwork was undertaken, the political-economy analysis is inferential, and all post-2011 population figures are treated as explicitly labelled projections rather than established facts.

## 4 Constitutional and legal architecture

### 4.1 Foundational constitutional provisions

The delimitation function rests on several constitutional provisions. Article 82 provides that after each census Parliament must by law readjust the allocation of Lok Sabha seats among the states and the division of each state into parliamentary constituencies; Article 170 imposes the same requirement for state legislative assemblies. Articles 330 and 332 require that seats be reserved for the Scheduled Castes and Scheduled Tribes in the Lok Sabha and the state assemblies in proportion to their populations. Together these provisions mandate delimitation as a census-linked, population-proportional process and establish a constitutional commitment to equitable representation (Maheshwari, 2000). Article 327 empowers Parliament to legislate on elections to either House and to the state legislatures, providing the statutory basis for the Delimitation Acts. Article 81 fixes the constitutional ceiling on Lok Sabha membership, currently 550 elected Members which any proposal to expand the House must address (De Santo and Le Maux, 2023).

### 4.2 The 1976 freeze: the 42nd constitutional amendment

The 42nd Amendment, passed during the Emergency, added provisions to Articles 82 and 170 freezing the total number of parliamentary and assembly seats assigned to each state at post-1971 levels until the first census after 2000 (Sivaramakrishnan, 1997). The stated justification was to avert the loss of seats for states that had successfully implemented family-planning programmes, recognising that a strict application of OPOV could create perverse incentives for demographic governance (Vaishnav and Hintson, 2019; Narain, 2021). The demographic reasoning had some validity: between 1951 and 1971 the fertility of states such as Kerala had already begun to fall, raising a legitimate concern that a purely population-based Lok Sabha would progressively disadvantage the more developed southern states (Bose, 1996; James and Sekher, 2023). But the Amendment’s indefinite extension has converted a prudent short-term measure into a significant constitutional deficiency (Visaria, 2000; Mishra, 2014).

### 4.3 The 84th and 87th amendments (2001–2003)

The 84th Amendment of 2001 extended the inter-state seat freeze until the first census after 2026 while permitting the internal alteration of constituency boundaries within states. The 87th Amendment of 2003 required the use of 2001 Census data for that internal readjustment and for determining reserved seats (Bhavnani and Kumar, 2021). This framework enabled the delimitation carried out under the Delimitation Act, 2002, which introduced new constituency maps based on the 2001 Census in February 2008 for most states (Boone and Wahman, 2015). The choice of 2026 as the freeze’s expiry was not arbitrary (McMillan, 2001): it was designed to allow two further census cycles to pass before the politically sensitive question of inter-state reallocation, a calculated postponement that successive administrations perpetuated through institutional inertia (Varshney, 2000).

### 4.4 The 106th amendment and the women’s reservation nexus (2023)

The 106th Amendment, the Nari Shakti Vandan Adhiniyam, 2023 guarantees a 33% reservation for women in the Lok Sabha, the state legislative assemblies, and the Delhi Legislative Assembly (Krook, 2009). The reservation applies to both general and SC/ST-reserved constituencies, with reserved seats to be rotated after each delimitation (Prillaman, 2023). Critically, the Amendment makes implementation dependent on the completion of the first post-commencement census and the following delimitation, forging a direct constitutional link between population enumeration, boundary adjustment, and gender-equity reform (Bhalotra et al., 2018; Spary, 2020). This has placed intense political pressure on the 2026 exercise. The government argues that proceeding on the 2011 Census, rather than awaiting 2026 data expected in 2027, is essential to enact women’s reservation in time for the 2029 general elections (Thomas, 2011). Critics counter, with Jensenius (2016) and Dovi (2007), that the legitimacy of gender quotas depends on the quality of their implementation, and that quotas built hastily on outdated demographic data detract from, rather than advance, the pursuit of gender justice.

## 5 Historical evolution of the delimitation commissions

### 5.1 The first delimitation commission (1952–53)

The first Delimitation Commission was constituted under the Delimitation of Parliamentary and Assembly Constituencies Order, 1951, chaired by Justice N. Chandrasekhara Aiyar. Using 1951 Census data, it increased the number of Lok Sabha seats from 489 to 494 (Singh, 2000). This exercise established the institutional template such as judicial chairmanship, associate membership of elected legislators, field visits, public representation, and gazette notification of final orders that successive commissions would follow (Gupta, 2015).

### 5.2 The second delimitation commission (1962–63)

The Second Delimitation Commission, using 1961 Census data, increased Lok Sabha seats from 494 to 522 (Ahuja, 1998; Sivaramakrishnan, 2015). This was the first exercise conducted after the States Reorganisation Act, 1956, which had redrawn state boundaries on linguistic lines, substantially enlarging the Commission’s task of delineating constituencies that respected both demographic proportionality and linguistic-administrative coherence (Alam and Sivaramakrishnan, 2015; Rudolph and Rudolph, 1987; Tillin, 2013).

### 5.3 The third delimitation commission (1972–76): the decisive exercise

The Third Delimitation Commission, chaired by Justice J. L. Kapur and using 1971 Census data, increased Lok Sabha seats from 522 to 542 (later 543 with Sikkim’s admission) and assembly seats from 3,771 to 3,997 (Patel and Sekher, 2026). This exercise is historically decisive because it fixed the inter-state seat allocations that have remained unchanged for over 50 years (Daxecker, 2020). The 1971 Census reflected the uneven population growth already beginning to favour northern states; by freezing these allocations, the 1976 amendment entrenched the political geography of a demographically divergent India as the permanent framework for parliamentary representation (Hussain, 2008; Elden, 2010). The forward-looking implications of that entrenchment, the projected seat shift once the freeze lifts are analysed as explicit scenarios, and are not treated here as historical fact.

### 5.4 The fourth delimitation commission (2002–08)

The Fourth Delimitation Commission, chaired by Justice Kuldip Singh, carried out the most extensive internal redrawing of constituency boundaries since 1973, using 2001 Census data as required by the 87th Amendment (Iyer and Reddy, 2013). The orders, effective from 19 February 2008, substantially changed the structure and demographic balance of many parliamentary and assembly seats and revised SC/ST reservations, while leaving the inter-state seat allocation frozen. Four northeastern states (Assam, Arunachal Pradesh, Manipur, and Nagaland) were excluded by separate presidential orders on security grounds, and Jharkhand’s orders were suspended until 2026 under Section 10B of the Delimitation Act, 2002 (Bhavnani, 2017).

## 6 Recent delimitation exercises: Jammu & Kashmir (2022) and Assam (2023)

The two most recent delimitations offer the closest available evidence of how a nationwide 2026 exercise might unfold. Read together across four dimensions, they are not merely recent illustrations but sources of specific propositions that feed forward into the reform proposals (Table 1).

**Table 1:** Structured comparison of the Jammu & Kashmir (2022) and Assam (2023) delimitation exercises across six analytical dimensions.

Dimension	Jammu & Kashmir (2022)	Assam (2023)
Legal foundation	J&K Reorganisation Act, 2019, applying the Delimitation Act, 2002	Representation of the People Act, 1950 (special provision for Assam)
Conducting body	A dedicated Delimitation Commission (chaired by Justice Ranjana Desai)	The Election Commission of India
Census reference year	2011	2001
Criteria beyond raw population	Physical features, communication, and public convenience under s. 9(1) of the 2002 Act; terrain and connectivity weighted	Administrative-unit boundaries following the December 2022 district reorganisation
Consultative procedure	Associate members and public sittings, but centrally directed after the abrogation of Article 370	ECI draft, objections, and hearings, but preceded by a contested district reorganisation
Reserved-seat effect	ST-reserved seats introduced for the first time	ST-reserved provision revised; boundary changes touched minority-concentration seats
Principal political reading	Rebalancing of Jammu and Kashmir divisions; contested as advantaging the incumbent	Internal boundaries redrawn without changing totals; effective weight of concentrated minorities reduced

### 6.1 Jammu & Kashmir (2022)

The Union Territory of Jammu and Kashmir underwent its most significant delimitation since 2008, following reorganisation under the J&K Reorganisation Act, 2019, and the repeal of Article 370. A Delimitation Commission constituted under the Delimitation Act, 2002, chaired by Justice Ranjana Prakash Desai, was tasked with defining assembly and parliamentary seats in accordance with the 2019 Act and the 2011 Census. In the final allocation, the Jammu region received 43 of the 90 assembly constituencies and the Kashmir region 47, a distribution that granted Jammu a share of seats somewhat above its share of population, which the Commission justified by reference to the geographical, administrative, and connectivity criteria permitted by Section 9(1) of the Delimitation Act, 2002. The exercise reserved nine assembly constituencies for Scheduled Tribes for the first time in the territory’s history, a reform facilitated by the revocation of Article 370. Because the structure of reserved-constituency boundaries shapes the substantive representation of marginalised communities over the long term (Jensenius, 2016), the J&K reservation warrants close attention as an institutional model for the nationwide process (Pande, 2003; Krishnan, 2007).

### 6.2 Assam (2023)

The Election Commission of India notified the delimitation of Assam on 11 August 2023, the first significant redrawing of the state’s boundaries in nearly 50 years. The total of 126 assembly and 14 Lok Sabha seats were unchanged, but boundaries were extensively modified and constituencies renamed. Of more than 1,200 public submissions, roughly 45% were incorporated into the final order. The exercise drew controversy over the use of the 2001 Census as its demographic base, which critics argued omitted the population change recorded in 2011 from the boundary calculations (Election Commission of India, 2023). Studies of malapportionment’s effect on economic development, alongside Iyer and Reddy’s (2013) work on incumbency effects in the 2002 exercise, supply a framework for assessing Assam: both show that redistricting produces measurable downstream effects on resource distribution, governance outcomes, and political trust (Besley and Burgess, 2002; Svaleryd, 2009). Consistent with this, Kukreja (2024) finds that districts gaining representation in state legislative assemblies exhibit increased public trust in political institutions, a result that bolsters the democratising potential of a well-executed delimitation, and, conversely, warns that perceived procedural irregularity of the kind alleged in Assam can produce the opposite effect (Jaffrelot, 2019).

### 6.3 The sociological dimension: caste arithmetic, minority concentration, and communities of interest

Beneath the state-level seat counts lie three sociological consequences that the preceding cases bring into view. The first is a change in the caste and community composition of individual constituencies. On Chandra’s (2004) logic, a party dependent on a particular caste bloc succeeds where that bloc is numerically pivotal within the seat; a boundary revision that disperses the bloc, or submerges it within a larger unit, can dissolve a winning coalition without any change in the underlying population. A 2026 exercise conducted after a five-decade freeze and, on the expansion scenarios, drawing a very large number of new boundaries at once would reset this arithmetic across the country simultaneously rather than incrementally. The relocation of SC- and ST-reserved constituencies compounds the effect: because reservation attaches to the units with the highest reserved-population share at each delimitation, seats shift geographically, and with them the map of where marginalised-group candidates can stand (Jensenius, 2017). Whether this advances substantive representation is contested (Jensenius, 2016; Zacharias and Vakulabharanam, 2011; Ziegfeld, 2016), so the redistribution should be read as a change in political opportunity structure, not an automatic gain.

The second is the dilution risk facing geographically concentrated ethno-religious minorities, whose translation of numbers into representation depends entirely on how boundaries are drawn, the same structural problem documented for racial minorities elsewhere (Lublin, 2020). Both of the present cases illustrate it: in Assam, the district reorganisation that preceded delimitation reshaped the units defining constituency boundaries, with consequences for the effective weight of concentrated minority populations; in Jammu and Kashmir, the rebalancing of seats between divisions altered the relative weight of the Muslim-majority Valley. Malapportionment and boundary manipulation are not politically neutral in their effects: Daxecker (2020) links unequal representation to election violence in India, the acute end of a spectrum whose ordinary form is the quiet marginalisation of concentrated minority electorates.

The third is the fracturing of communities of interest. Equalising population across constituencies can sever the local ties of caste settlement, linguistic region, and customary association through which representation is actually experienced; territory here is not a neutral container but a social relation (Elden, 2010). In a single-shot 2026 delimitation drawing many boundaries at once, the aggregate disruption to these ties is qualitatively different from the incremental adjustment of a routine boundary review. This is not an argument against equalisation, which OPOV requires; it is an argument for the procedural safeguards, community-of-interest criteria, transparent consultation, and restraint on pre-delimitation administrative-boundary changes.

## 7 The 2026 crossroads: political economy of a historic threshold

### 7.1 The proposed 50% seat expansion

The government’s plan to increase Lok Sabha seats by roughly 50%, from 543 to about 816, is a structural response to the politics of the North–South divide. By expanding seats broadly in line with each state’s current share, it aims to preserve every state’s existing delegation while allowing proportional adjustment (Poterba, 1994). De Santo and Le Maux (2023), studying optimal legislature size, note that legislatures tend to scale with the cube root of population implying an optimal Lok Sabha of roughly 750–800 members, broadly consistent with the proposed enlargement (Mebane and Sekhon, 2004).

The demographic base of the proposal is contested. Building on 2011 Census data while 2026 data will not be available until 2027 embeds a fifteen-year lag in the representational calculation (Alam and Sivaramakrishnan, 2015). Patel and Sekher (2026) show that uneven fertility has intensified since 2011, so projections based on 2011 data will understate the population share of northern states and overstate that of the south. A delimitation built on 2011 data would therefore embed demographic inaccuracy into the representational framework from the outset.

The illustrative model reported in the Supplementary material makes these stakes concrete. Under a fixed 543-seat house on the 2011 base, strict population apportionment costs the five southern states 17 seats and hands the northern bloc 26; under the proposed 816-seat house, no region loses in absolute terms, but the relative shares still move north. The scenario matrix below summarises the seat position of the two blocs under each combination of base, house size, and allocation rule, with the 2026-projected rows to be completed once the model is re-run on the National Commission on Population projections (Table 2).

**Table 2:** Seat allocation scenarios for southern and northern blocs under varying census base years, house sizes, and apportionment rules.

Scenario	South	North
Current (frozen, 543)	129	243
2011 base-543-pure population	112	269
2011 base-543-70/30 formula	127	235
2011 base-816-pure population	170	406
2011 base-816-70/30 formula	191	354
2026-projected-816-pure population	To compute	To compute
2026-projected-816-70/30 formula	To compute	To compute

### 7.2 The North–South federal fault line and its fiscal foundations

The North–South dimension of the debate represents what Srinivasulu (2024) characterises as the tension between a centralised, majoritarian “view of India from above” and a pluralistic, developmentally diverse “view of India from the regions” (Sivaramakrishnan, 2000). Southern chief ministers and much of the opposition have consistently pressed the “population-control penalty” argument, casting delimitation as an existential threat to the political weight of the south (Vaishnav and Hintson, 2019; Suri, 2004).

That resistance is more intense than a seat count alone would explain, because delimitation is the representational face of a broader fiscal-federal grievance, and the two reinforce each other. The southern objection is to a double penalty. Having reduced fertility and raised incomes, these states already receive a declining share of central tax devolution, successive Finance Commission formulas weight population heavily, so a state that curbed its population is transferred proportionately less and they now face losing legislative weight for the same demographic success. Relative-income data sharpen the sense of a subsidy flowing north: the Economic Advisory Council to the Prime Minister (2024) records the southern states well above, and the large northern states well below, national per-capita income, so devolution transfers resources from the former to the latter even as representation is set to move in the opposite direction. The Sixteenth Finance Commission (2026), reporting as the freeze expires, has itself begun shifting transfers toward performance and economic-contribution criteria, an implicit acknowledgement of the tension. Read as political economy, delimitation and devolution form a single bargain: representation determines who controls the centre that redistributes revenue, so a population-driven seat shift is a matter not only of voice but of the future division of fiscal resources. A settlement confined to the Lok Sabha, without attention to the fiscal and second-chamber dimensions, is therefore unlikely to hold. The present author’s view is that uniform or degressive representation of the states in the Rajya Sabha, on the model of the United States Senate, would offer a constitutionally cleaner counter-majoritarian mechanism than distorting the first chamber.

### 7.3 The Census-Base controversy

The disputed choice between the 2011 and 2026 censuses as the demographic base exemplifies the tension between political expediency and democratic accountability. McMillan’s (2001) analysis used 2001 Census projections to estimate the seat allocations that would have obtained had the freeze lifted; Vaishnav and Hintson (2019) revised this using 2011 projections. The divergence between their figures and the larger projected shift reported by Patel and Sekher (2026) is precisely a base-year effect: the projected 2026 base widens the northern gain because the high-fertility states grow fastest between 2011 and 2026. Presented within the single scenario matrix of Section 7.1, the apparent inconsistency becomes a demonstration of base-year sensitivity and both analyses agree that the severity of malapportionment worsens with each delayed census cycle.

The constitutional stakes are correspondingly high. A delimitation freeze whether legislated or produced by executive inaction sits in tension with the guarantee of equality under Article 14, an argument strengthened by the established precedents of *Baker v. Carr* (1962) and *Reynolds v. Sims* (1964) and by the comparative constitutional-law literature on effective voting rights (Norris, 2004; Rossiter et al., 2009). Should the Supreme Court of India accept this reasoning, a government preference for delimitation on the 2011 Census could invite direct constitutional challenge.

### 7.4 Women’s reservation and the delimitation timeline

The implementation of the Nari Shakti Vandan Adhiniyam, 2023, through delimitation has bound together two conflicting governance imperatives (Bhalotra et al., 2018). The women’s-reservation rationale gives the government a strong justification for immediate delimitation; but the same conditionality means that any postponement is cast as a delay in gender justice, generating pressure for a speed that could undermine the legitimacy of the process (Mansbridge, 2009). Research on gender quotas—Jensenius (2016) on India’s panchayat reservations, Spary (2020) on national quotas indicates that the representational benefits of reservation depend heavily on the design and execution of the system rather than its mere existence (Thomas, 2011; Meier, 2008). A lottery-based random selection of reserved constituencies from existing Lok Sabha seats, considered during the Bill’s passage, would permit implementation without extensive delimitation, decoupling the two reforms so each can proceed on its own timeline (Tripp and Kang, 2008; Krook, 2009).

### 7.5 Comparative Federal Perspectives

Every mature federation faces India’s dilemma, and each resolves it with a structural device almost always by building federal equity into a second chamber or into statutory floors, rather than by distorting population-based apportionment in the first chamber. The comparison locates India’s problem precisely (Table 3).

**Table 3:** Federal mechanisms offsetting populationbased apportionment in five mature federations and their relevance to India’s delimitation reform.

Federation	Device that offsets population	Bearing on India
Australia	Senate seats equal across states; House “as nearly as practicable, twice the number of senators” (s. 24 “nexus”); a guaranteed minimum of five House seats per Original State	The nexus ties any House expansion to Senate expansion—a caution directly relevant to India’s proposed enlargement; the five-seat floor is a statutory analogue to a seat-cushion
Canada	Population-based Commons formula (s. 51, Constitution Act 1867) tempered by the senatorial clause, the grandfather clause, and the representation rule	A worked model of population apportionment plus explicit floors that stop slower-growing units from losing seats close to India’s expansion-with-protection logic
Germany	Bundestag population-based (MMP, Sainte-Laguë); federal equity sits in the Bundesrat, where Länder hold three to six votes on a degressive scale (Art. 51(2) Basic Law)	Shows the counterweight belongs in the second chamber exactly what India’s roughly population-based Rajya Sabha fails to provide
United States	House apportioned by strict population equality (*Baker v. Carr*; *Reynolds v. Sims*); Senate gives every state two seats regardless of size	The bicameral bargain: strict equality in one chamber is politically tolerable only because the other guarantees federal equity
European Union	Parliament seats allocated by degressive proportionality (Art. 14(2) TEU)	Confirms that non-population weighting, where adopted, is constitutionalised at the highest level supporting the amendment conclusion

The comparison yields the paper’s central diagnostic claim. In Australia, Canada, Germany, and the United States, the pressure that population growth places on representation is released through a second chamber (or statutory floor) that is deliberately not population-proportional. India lacks this release valve: the Rajya Sabha is allocated to states roughly in proportion to population and so reproduces, rather than offsets, the Lok Sabha’s bias. The entire burden of the federal-equity claim therefore falls on Lok Sabha delimitation which is why the 2026 exercise is so combustible, and why the two realistic remedies are either to weight the first chamber or to rebuild the second.

## 8 Challenges and reform proposals

### 8.1 Malapportionment: the case for a weighted equity formula

The most structurally difficult task is designing an inter-state allocation formula that honours both OPOV and federal equity. Balinski and Young (2001) demonstrate that no formula can simultaneously satisfy every normative criterion for proportional allocation; the question is which trade-off is constitutionally and politically acceptable.

The formula proposed here weights each state’s allocation base as a combination of population and development. Let each state *i* have census population *Pᵢ* and a development score *Dᵢ* (rewarding high literacy and low fertility, the “demographic governance performance” the 1976 freeze sought to protect). Writing the population share *pᵢ = Pᵢ/*Σ*P* and a population-anchored development share *dᵢ*, the allocation base is *bᵢ = w·pᵢ + (1 − w)·dᵢ*, with *w* = 0.70; seats follow from applying the Webster method to *bᵢ·H*, subject to a one-seat floor. The full model, data, and a sensitivity analysis across weightings from 100/0 to 60/40 are reported in the Supplementary material. Its central finding substantiates the choice of 70/30: under a fixed 543-seat house, strict population costs the south 17 seats, and the 70/30 weighting cushions all but two of them (about 88%) while neutralising the northern windfall, whereas a heavier 60/40 weight over-corrects turning the south into a net gainer and stripping the north of seats from its current baseline. Seventy-thirty is thus the lowest development weight that neutralises the southern penalty without over-correcting.

Two qualifications are essential. First, the constitutional test: Article 81(2)(a) requires that the ratio of a state’s seats to its population be “so far as practicable, the same for all States.” That language authorises administrative adjustments, not a systematic 30% reweighting toward non-population criteria; a formula of this magnitude therefore could not be adopted by an ordinary Delimitation Act and would require a constitutional amendment. Second, the comparative anchors point the same way: Canada’s senatorial and grandfather clauses (s. 51, Constitution Act 1867) and the European Parliament’s degressive proportionality (Art. 14(2) TEU) both weight representation away from strict population, and both are constitutionalised rather than statutory (Handley and Grofman, 2008). Germany illustrates the same lesson from the other side, its Bundestag is population-based, and its federal-equity weighting lives in the Bundesrat.

### 8.2 Process integrity: consultation, transparency, and the manipulation risk

Iyer and Reddy (2013) found that, although the 2002 exercise was largely neutral, advisory members were able to influence boundary decisions in Andhra Pradesh and Rajasthan to protect incumbents. An enhanced anti-manipulation protocol, a statutory moratorium on administrative-boundary changes within 18 months of a notified delimitation, mandatory publication of draft proposals in all scheduled languages, a minimum ninety-day public-comment period, and written reasons for accepting or rejecting each representation would strengthen the Commission’s credibility without requiring constitutional amendment. Both Bhavnani (2017) and Daxecker (2020) show that perceived process integrity strongly shapes political trust and participation (Narain, 2021), and Kukreja’s (2024) finding on the redistricting–trust relationship confirms that improving process quality is not merely procedurally desirable but consequential for governance outcomes (Besley and Burgess, 2002).

### 8.3 Census infrastructure: the case for a census timeliness act

India’s 2021 Census was postponed indefinitely because of the Covid-19 pandemic, leaving the country reliant on 2011 data for nearly 5 years and precipitating the current dilemma over the delimitation deadline (National Commission on Population, 2020). James and Sekher (2023) document the cascading governance failures that follow census delays, affecting political representation, welfare targeting, fiscal devolution, and infrastructure planning (Mishra, 2014; Visaria, 2000). Parliament should consider a Census Timeliness Act requiring the census to be conducted within 90 days of its scheduled commencement, with independent oversight of the process.

### 8.4 GIS modernisation and technological transparency

Previous commissions worked largely through paper-based methods, with limited use of Geographic Information System (GIS) technology for boundary visualisation, demographic mapping, or public engagement (Rajagopal, 2005). Handley and Grofman (2008) highlight open-source GIS as a key factor in both process quality and public trust in boundary commissions. India’s digital public infrastructure such as Aadhaar, the Geospatial Data Promotion and Utilisation Policy, and the drone-based village mapping of the SVAMITVA scheme provides the technological basis for a modern, evidence-driven, and publicly contestable delimitation process (Elden, 2010).

### 8.5 Decoupling women’s reservation from delimitation

Making women’s reservation contingent on delimitation creates incentives to hurry, and potentially to lower the quality of, the boundary-drawing (Prillaman, 2023). A legislative amendment to the Nari Shakti Vandan Adhiniyam, 2023, authorising the Election Commission to identify reserved constituencies by lottery-based random selection from existing Lok Sabha seats, would honour the constitutional mandate for gender equality while allowing delimitation to proceed on a timetable that permits the use of 2026 Census data and full consultation (Teele et al., 2018). This aligns with Jensenius’s (2016) finding that the representational benefits of gender quotas hold regardless of the particular selection process used (Chattopadhyay and Duflo, 2004).

### 8.6 Implementation, feasibility, and alternatives

Three obstacles condition any reform. The first is constitutional: a weighted-equity formula of material size requires an amendment under Article 368, and because it touches the federal distribution of power it may attract the requirement of ratification by half of the state legislatures. The second is institutional: the reform presupposes a completed 2026 census, a Commission insulated from executive influence over the administrative boundaries that feed it, and the data capacity to apply a multi-indicator formula transparently conditions the Assam experience shows cannot be assumed. The third is political, and it is decisive: the northern states that gain under strict population have both the parliamentary majority to enact a population-favouring settlement and little incentive to concede a cushion, while the southern states that would benefit from one lack the numbers to compel it.

A balanced treatment requires stating the rival options at their strongest. Four plausible approaches emerge. Expansion with strict population and no cushion is the cleanest on OPOV grounds, if the house grows enough, no state loses seats absolutely, so on this view a cushion is an unnecessary distortion of equal suffrage but it addresses absolute loss without answering the relative shift of national power that is the actual grievance. Rebuilding the second chamber, by giving the Rajya Sabha the equal or degressive state representation it currently lacks, is arguably the most principled fix, since it protects equal suffrage in the first chamber while housing federal equity where comparative practice puts it; its weakness is the Rajya Sabha’s weaker powers and an equally heavy amendment burden. Phased or sunset delimitation would smooth the shock and let demographic convergence narrow the gap over time, at the cost of prolonging existing malapportionment. Continued freeze is the easiest to legislate and the hardest to defend, deepening the very malapportionment the exercise is meant to cure.

Against these, the paper’s position is deliberately conditional rather than absolute. Expansion should proceed, because preventing absolute loss is the necessary first step and the least contestable. Beyond that, the durable settlement is either a weighted first chamber or a rebuilt second chamber substitutes, both requiring amendment and the choice between them is as much political as constitutional. We favour the weighted formula for its directness and because it can be made transparent and rule-bound; but we acknowledge that second-chamber reform is the more principled route on equal-suffrage grounds, and that a phased path may be the only politically survivable one.

## 9 Conclusion

India’s post-2026 delimitation cannot be reduced to arithmetic; it is a choice about how a federation reconciles equal suffrage with federal equity, made harder by the absence of the second-chamber counterweight that other federations rely on. As this paper has argued through a five-lens framework and a mixed-method analysis including a reproducible allocation model and a comparison with four federations, the challenges of 2026 are fundamentally normative, requiring choices among competing constitutional values: between OPOV and federal equity, between implementation speed and demographic accuracy, and between gender justice and deliberative legitimacy. The existing literature, from McMillan’s (2001) foundational calculations through the Webster projections of Vaishnav and Hintson (2019) and Patel and Sekher (2026), demonstrates that the representational deficit has reached a point that cannot responsibly be postponed; findings from redistricting research of Kukreja (2024) on political trust, Iyer and Reddy (2013) on incumbency influence, Daxecker (2020) on malapportionment and violence show that the quality of the process matters as much as its execution.

The analysis yields five firm recommendations, in priority order. First, base the exercise on 2026 census data, since current data is the precondition for any legitimate apportionment. Second, expand the Lok Sabha as proposed, because enlargement prevents the absolute seat losses that make reform zero-sum. Third, pair expansion with a federal-equity mechanism enacted by constitutional amendment, either a transparent, rule-bound weighted-equity formula in the Lok Sabha or a rebalanced Rajya Sabha rather than leaving the entire tension on population-based apportionment. Fourth, protect process integrity through a moratorium on administrative-boundary changes before delimitation and a guarantee of the Commission’s independence, supported by GIS-based transparency and a Census Timeliness Act. Fifth, implement the women’s reservation on a transparent timetable, so that gender justice is delivered by the reform rather than used to bargain for it. These steps will not dissolve the underlying North–South tension, which is demographic and fiscal as well as electoral; but they would convert a potentially destabilising reallocation of power into a constitutionally defensible one, befitting the world’s largest democracy.

## Data Availability

The original contributions presented in the study are included in the article/Supplementary material, further inquiries can be directed to the corresponding author.

## AppendixTable of Legislation and Cases

### Legislation

Constitution of India, Articles. 81, 82, 170, 327, 330, 332

Constitution (Forty-second Amendment) Act, 1976

Constitution (Eighty-fourth Amendment) Act, 2001

Constitution (Eighty-seventh Amendment) Act, 2003

Constitution (One Hundred and Sixth Amendment) Act, 2023 (Nari Shakti Vandan Adhiniyam)

Delimitation Act, 1952; Delimitation Act, 1962; Delimitation Act, 1972; Delimitation Act, 2002

Jammu and Kashmir Reorganisation Act, 2019

Commonwealth of Australia Constitution Act 1900, s. 24

Constitution Act, 1867 (Canada), ss. 51, 51A

Fair Representation Act, 2011 (Canada)

Basic Law for the Federal Republic of Germany, art. 51(2)

Treaty on European Union, Art. 14(2)

U.S. Constitution, Art. I, Section 2

### Cases

*Baker v. Carr*, 369 U.S. 186 (1962).

*Reynolds v. Sims*, 377 U.S. 533 (1964).
